# Editorialets

**Published:** 1906-11

**Authors:** 


### Editorialets.

Drs. Jesse M. Pace and Charles M. Rosser have been sworn in as members of the board of health of Dallas.

Dr. R. H. McHenry, of Mineral Wells, died October 13th. Dr. McHenry was formerly of San Angelo, and his remains were interred in that city.

For Sale.—$2500 unopposed country practice; nearest doctor ten miles. Collections 95 per cent; no boll weevil; good house, barn, office, private water system; many Germans. Price, $1600.

Dr. T. E. Boyer, Shive, Texas.

Dr. W. K. Fowler, of Mineral Wells, Texas, died at his home October 18th, ult., aged 28. Dr. Fowler was a native of Los Angeles, Cal. He served a short time in the U. S. Army in the Philippines. At the time of his death he was connected with the Mineral Wells Sanitarium.

"Which I wish to remark and my language is plain," there never was a time in the twenty-one years of the "Red Back's" little life when cash for subscriptions, new, renewals and delinquent, were more in order, or will be more timely and welcome. Every little bit helps. Please send along your little checks. *Do it now!*

Tabor Endorsed.—At a meeting of the Dallas County Medical Association, October 1, resolutions were unanimously adopted endorsing the recommendation that the incoming Governor continue in office the present State health officer, Dr. George R. Tabor, whose service has proved entirely satisfactory and reflects unusual credit on the medical profession.

Pasteur Institute Report.—Dr. Benjamin M. Worsham, Austin, superintendent of the State Pasteur Institute, reports that during the first fiscal year 81 patients were treated without the development of a single case of hydrophobia, and that during the

The usual combination of impoverished blood, nervous exhaustion, lack of digestive vigor yields most quickly to

GRAY'S GLYCERINE TONIC COMP.

It restores, nourishes, reconstructs; besides this, it is especially efficient in diseases of the Chest and Throat

THE PURDUE FREDERICK CO.,
298 Broadway, New York.

second year 245 patients were treated, 226 of whom came from Texas, with 2 deaths. The total fees collected were $2510 and the disbursements were $1039.36.

---

STATE HEALTH OFFICER'S REPORT.—In the biennial report of Dr. George R. Tabor, State health officer, he directs attention to the fact that four years ago 75 per cent of the populated counties of Texas were infected with smallpox, but that the disease was rapidly decreasing. Yellow fever has not appeared in Texas this year, and when it was prevalent in Louisiana last year it was kept out of Texas at a cost of less than $30,000. He bears testimony to the efficient services rendered by the Texas quarantine officers on the borders of Louisiana, Arkansas, and Indian Territory. The operating expenses of the department for two years amounted to $45,372, or less than for the previous biennium.

---

GOING FOR TYREE.—The Council on Pharmacy, A. M. O., have analyzed Tyree's Antiseptic powder and reported that it is not what it claims to be. In their report they say: "Mr. Tyree assumes the right to sell to physicians a preparation with a descriptive formula which he acknowledges is false, and that he presumes to use his own pleasure to the time when he will inform them of its true composition. * * * On April 4 Mr. Tyree was notified by the council that the composition of 'Tyree's Antiseptic Powder' did not correspond with the formula published by him. * * * Whether or not Mr. Tyree is justified in offering our profession a preparation as composed chiefly of borax and alum when in reality it is chiefly composed of boric acid and zinc sulphate, we leave physicians to judge. * * * In view of the fact that J. S. Tyree has given wide publicity to a formula which the preceding report has shown to be a deliberate misrepresentation of facts, it is recommended that the article be refused recognition by the Council on Pharmacy and Chemistry, and that this report be published in *The Journal of the American Medical Association.—Extracts of Report in Journal A. M. A.*

